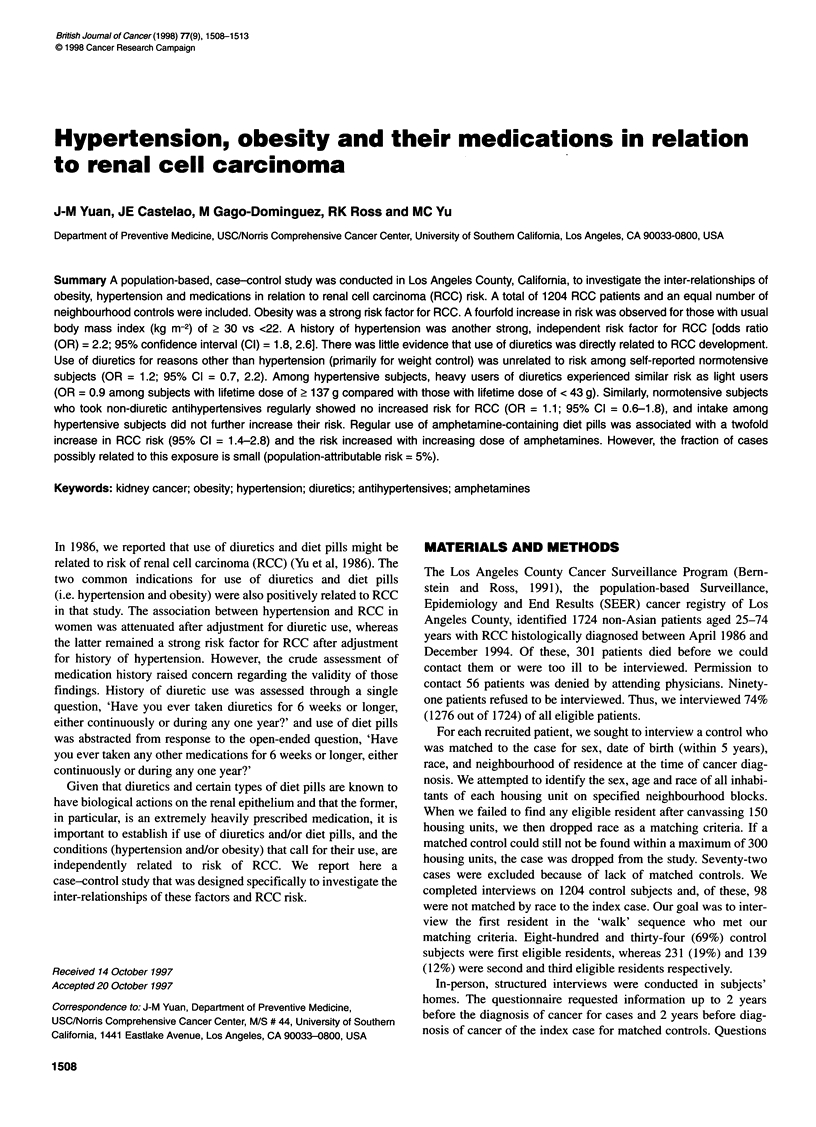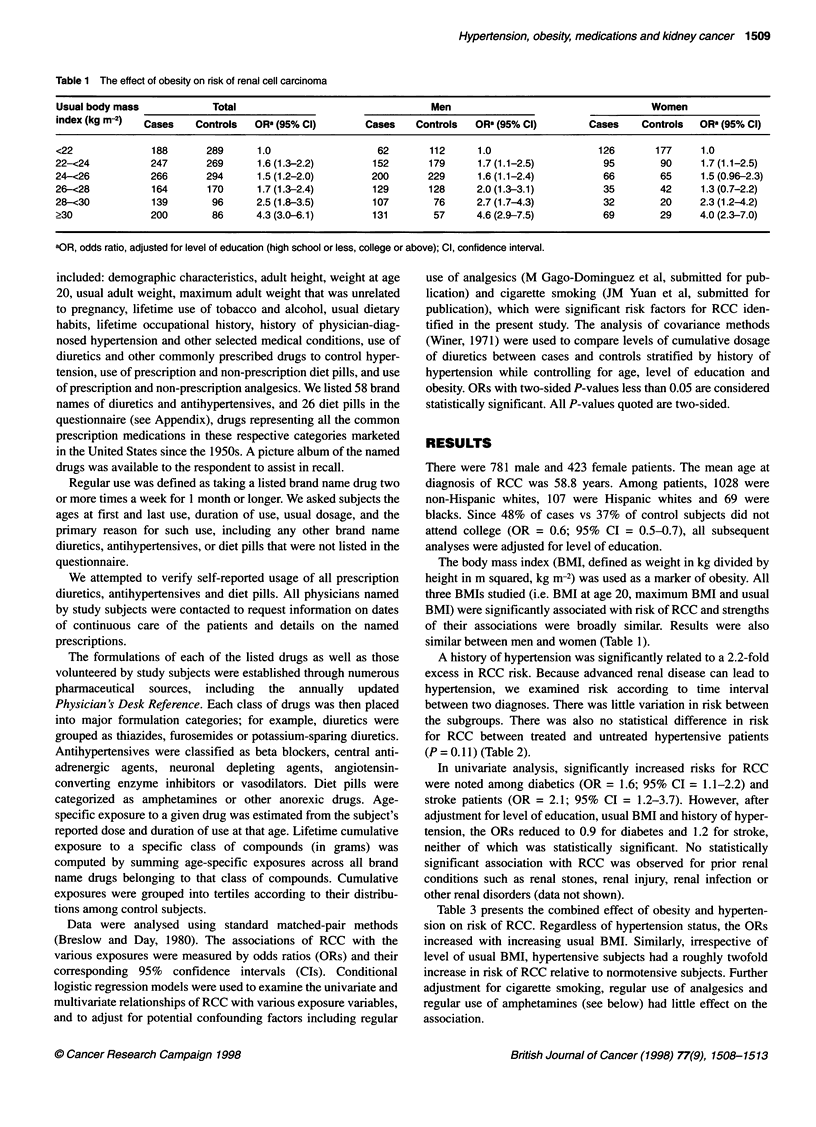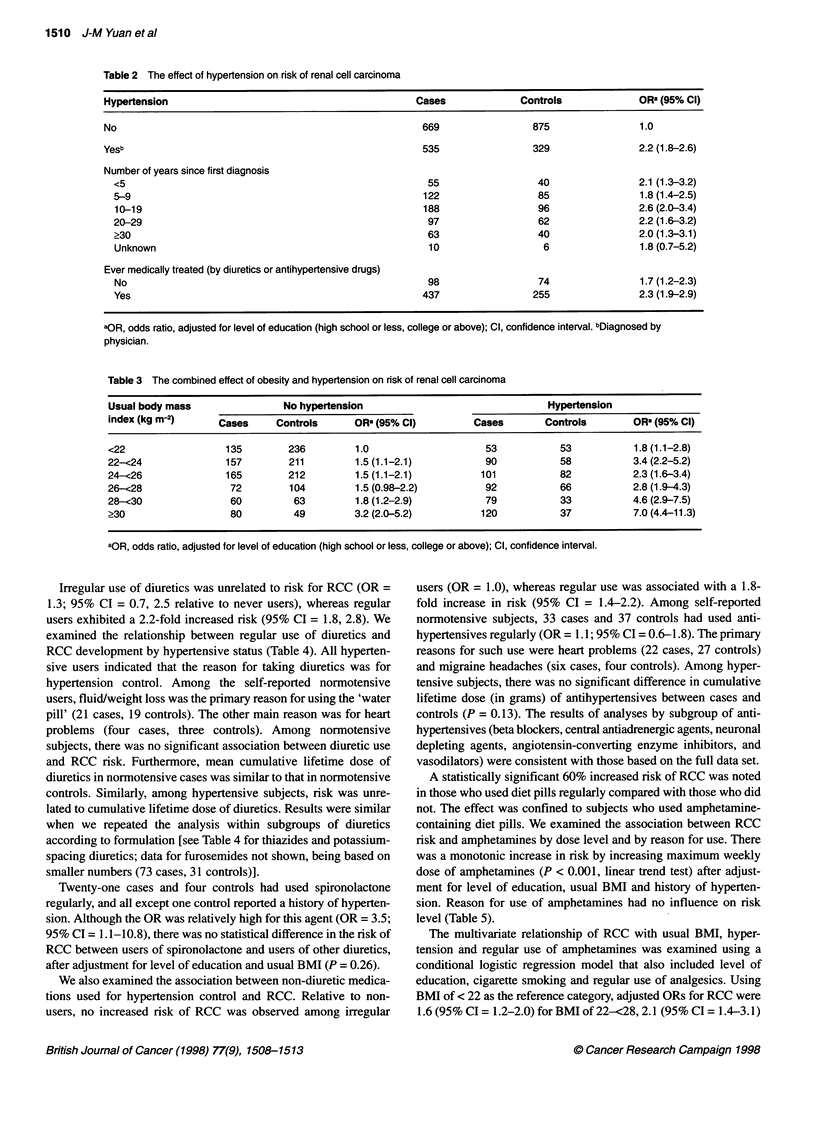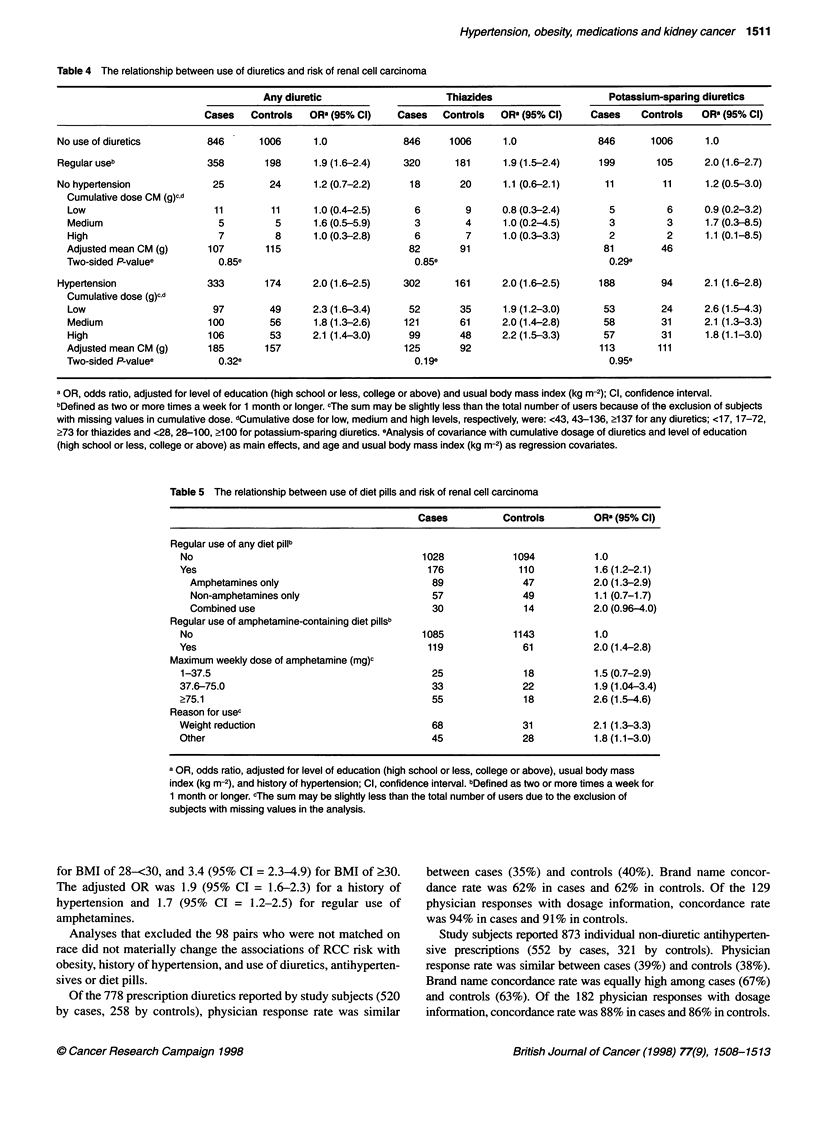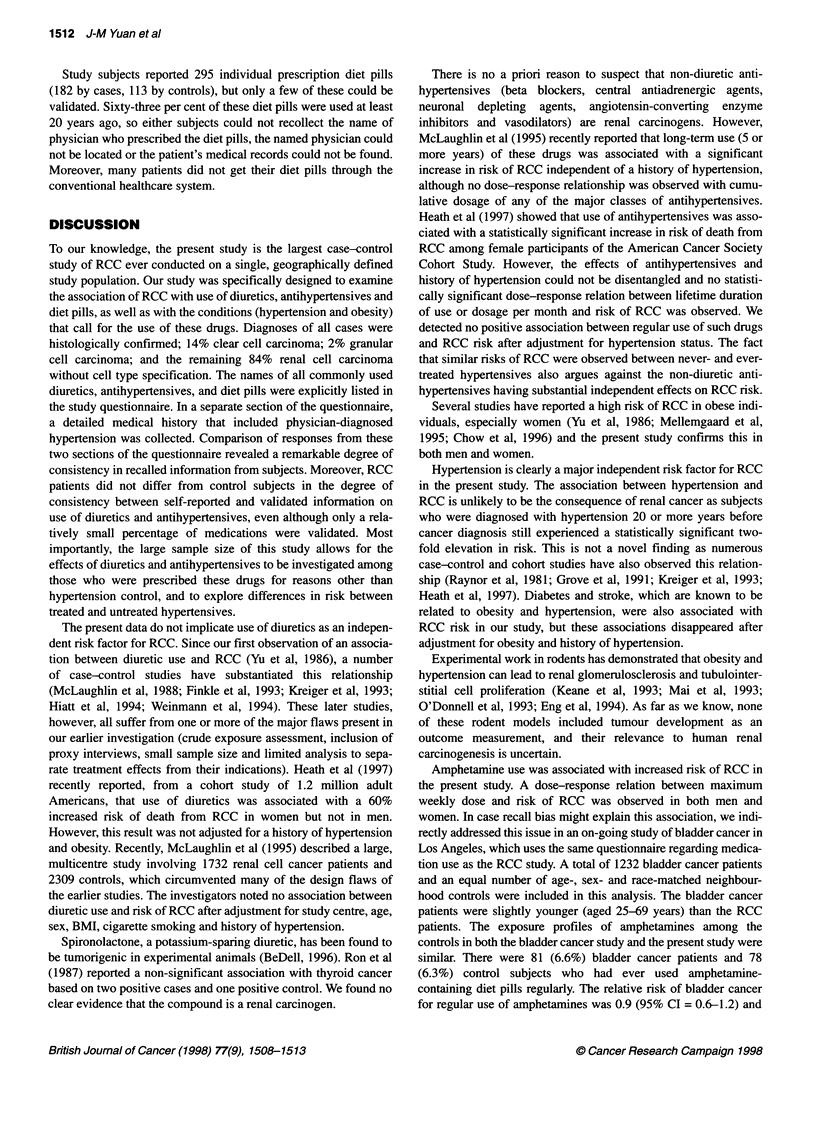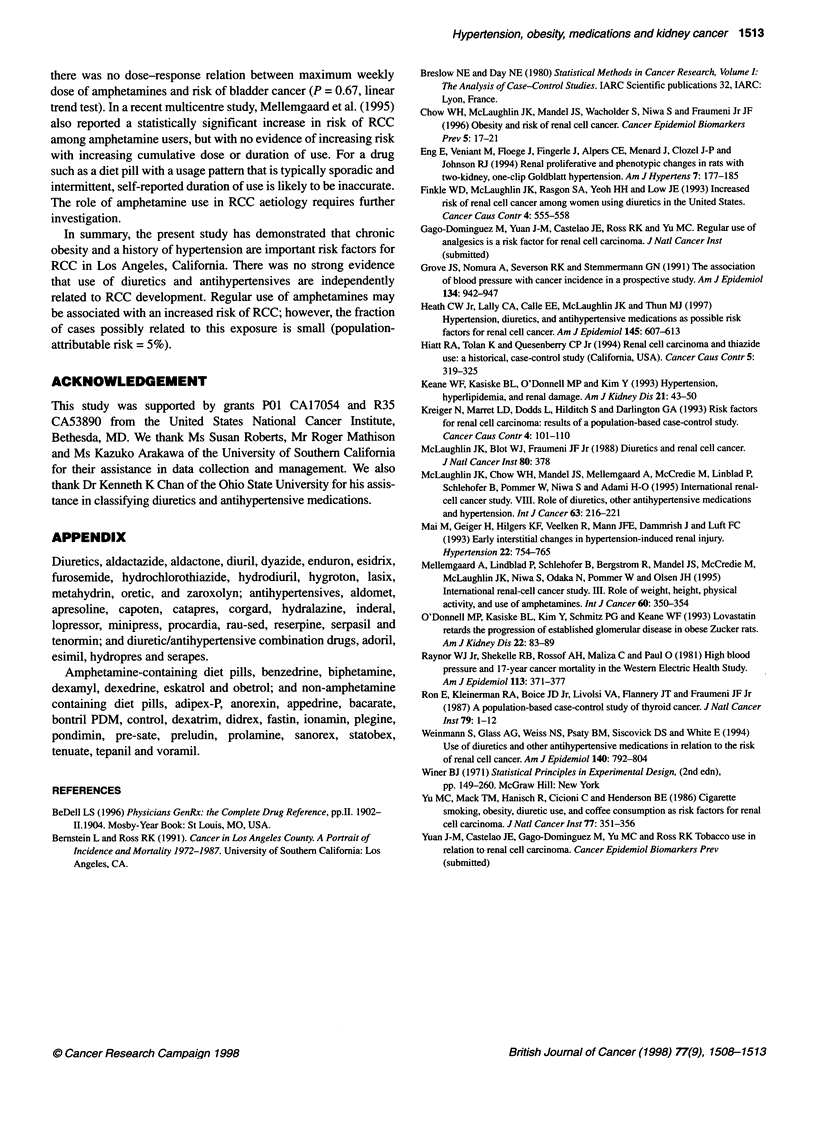# Hypertension, obesity and their medications in relation to renal cell carcinoma.

**DOI:** 10.1038/bjc.1998.248

**Published:** 1998-05

**Authors:** J. M. Yuan, J. E. Castelao, M. Gago-Dominguez, R. K. Ross, M. C. Yu

**Affiliations:** Department of Preventive Medicine, USC/Norris Comprehensive Cancer Center, University of Southern California, Los Angeles 90033-0800, USA.

## Abstract

A population-based, case-control study was conducted in Los Angeles County, California, to investigate the inter-relationships of obesity, hypertension and medications in relation to renal cell carcinoma (RCC) risk. A total of 1204 RCC patients and an equal number of neighbourhood controls were included. Obesity was a strong risk factor for RCC. A fourfold increase in risk was observed for those with usual body mass index (kg m(-2)) of > or = 30 vs < 22. A history of hypertension was another strong, independent risk factor for RCC [odds ratio (OR) = 2.2; 95% confidence interval (CI) = 1.8, 2.6]. There was little evidence that use of diuretics was directly related to RCC development. Use of diuretics for reasons other than hypertension (primarily for weight control) was unrelated to risk among self-reported normotensive subjects (OR = 1.2; 95% CI = 0.7, 2.2). Among hypertensive subjects, heavy users of diuretics experienced similar risk as light users (OR = 0.9 among subjects with lifetime dose of > or = 137 g compared with those with lifetime dose of < 43 g). Similarly, normotensive subjects who took non-diuretic antihypertensives regularly showed no increased risk for RCC (OR = 1.1; 95% CI = 0.6-1.8), and intake among hypertensive subjects did not further increase their risk. Regular use of amphetamine-containing diet pills was associated with a twofold increase in RCC risk (95% CI = 1.4-2.8) and the risk increased with increasing dose of amphetamines. However, the fraction of cases possibly related to this exposure is small (population-attributable risk = 5%).


British Journal of Cancer (1998) 77(9), 1508–1513
© 1998 Cancer Research Campaign

### Hypertension, obesity and their medications in relation to renal cell carcinoma

**J-M Yuan, JE Castelao, M Gago-Dominguez, RK Ross and MC Yu**

Department of Preventive Medicine, USC/Norris Comprehensive Cancer Center, University of Southern California, Los Angeles, CA 90033-0800, USA

**Summary** A population-based, case–control study was conducted in Los Angeles County, California, to investigate the inter-relationships of obesity, hypertension and medications in relation to renal cell carcinoma (RCC) risk. A total of 1204 RCC patients and an equal number of neighbourhood controls were included. Obesity was a strong risk factor for RCC. A fourfold increase in risk was observed for those with usual body mass index (kg $m^{-2}$) of ≥ 30 vs <22. A history of hypertension was another strong, independent risk factor for RCC [odds ratio (OR) = 2.2; 95% confidence interval (CI) = 1.8, 2.6]. There was little evidence that use of diuretics was directly related to RCC development. Use of diuretics for reasons other than hypertension (primarily for weight control) was unrelated to risk among self-reported normotensive subjects (OR = 1.2; 95% CI = 0.7, 2.2). Among hypertensive subjects, heavy users of diuretics experienced similar risk as light users (OR = 0.9 among subjects with lifetime dose of ≥ 137 g compared with those with lifetime dose of < 43 g). Similarly, normotensive subjects who took non-diuretic antihypertensives regularly showed no increased risk for RCC (OR = 1.1; 95% CI = 0.6–1.8), and intake among hypertensive subjects did not further increase their risk. Regular use of amphetamine-containing diet pills was associated with a twofold increase in RCC risk (95% CI = 1.4–2.8) and the risk increased with increasing dose of amphetamines. However, the fraction of cases possibly related to this exposure is small (population-attributable risk = 5%).

**Keywords:** kidney cancer; obesity; hypertension; diuretics; antihypertensives; amphetamines

In 1986, we reported that use of diuretics and diet pills might be related to risk of renal cell carcinoma (RCC) (Yu et al, 1986). The two common indications for use of diuretics and diet pills (i.e. hypertension and obesity) were also positively related to RCC in that study. The association between hypertension and RCC in women was attenuated after adjustment for diuretic use, whereas the latter remained a strong risk factor for RCC after adjustment for history of hypertension. However, the crude assessment of medication history raised concern regarding the validity of those findings. History of diuretic use was assessed through a single question, 'Have you ever taken diuretics for 6 weeks or longer, either continuously or during any one year?' and use of diet pills was abstracted from response to the open-ended question, 'Have you ever taken any other medications for 6 weeks or longer, either continuously or during any one year?'

Given that diuretics and certain types of diet pills are known to have biological actions on the renal epithelium and that the former, in particular, is an extremely heavily prescribed medication, it is important to establish if use of diuretics and/or diet pills, and the conditions (hypertension and/or obesity) that call for their use, are independently related to risk of RCC. We report here a case–control study that was designed specifically to investigate the inter-relationships of these factors and RCC risk.

*Received 14 October 1997*
*Accepted 20 October 1997*

*Correspondence to:* J-M Yuan, Department of Preventive Medicine, USC/Norris Comprehensive Cancer Center, M/S # 44, University of Southern California, 1441 Eastlake Avenue, Los Angeles, CA 90033–0800, USA

### MATERIALS AND METHODS

The Los Angeles County Cancer Surveillance Program (Bernstein and Ross, 1991), the population-based Surveillance, Epidemiology and End Results (SEER) cancer registry of Los Angeles County, identified 1724 non-Asian patients aged 25–74 years with RCC histologically diagnosed between April 1986 and December 1994. Of these, 301 patients died before we could contact them or were too ill to be interviewed. Permission to contact 56 patients was denied by attending physicians. Ninety-one patients refused to be interviewed. Thus, we interviewed 74% (1276 out of 1724) of all eligible patients.

For each recruited patient, we sought to interview a control who was matched to the case for sex, date of birth (within 5 years), race, and neighbourhood of residence at the time of cancer diagnosis. We attempted to identify the sex, age and race of all inhabitants of each housing unit on specified neighbourhood blocks. When we failed to find any eligible resident after canvassing 150 housing units, we then dropped race as a matching criteria. If a matched control could still not be found within a maximum of 300 housing units, the case was dropped from the study. Seventy-two cases were excluded because of lack of matched controls. We completed interviews on 1204 control subjects and, of these, 98 were not matched by race to the index case. Our goal was to interview the first resident in the 'walk' sequence who met our matching criteria. Eight-hundred and thirty-four (69%) control subjects were first eligible residents, whereas 231 (19%) and 139 (12%) were second and third eligible residents respectively.

In-person, structured interviews were conducted in subjects' homes. The questionnaire requested information up to 2 years before the diagnosis of cancer for cases and 2 years before diagnosis of cancer of the index case for matched controls. Questions

**Table 1** The effect of obesity on risk of renal cell carcinoma

| Usual body mass index (kg m⁻²) | Total | | | Men | | | Women | | |
|---|---|---|---|---|---|---|---|---|---|
| | Cases | Controls | OR[a] (95% CI) | Cases | Controls | OR[a] (95% CI) | Cases | Controls | OR[a] (95% CI) |
| <22 | 188 | 289 | 1.0 | 62 | 112 | 1.0 | 126 | 177 | 1.0 |
| 22–<24 | 247 | 269 | 1.6 (1.3–2.2) | 152 | 179 | 1.7 (1.1–2.5) | 95 | 90 | 1.7 (1.1–2.5) |
| 24–<26 | 266 | 294 | 1.5 (1.2–2.0) | 200 | 229 | 1.6 (1.1–2.4) | 66 | 65 | 1.5 (0.96–2.3) |
| 26–<28 | 164 | 170 | 1.7 (1.3–2.4) | 129 | 128 | 2.0 (1.3–3.1) | 35 | 42 | 1.3 (0.7–2.2) |
| 28–<30 | 139 | 96 | 2.5 (1.8–3.5) | 107 | 76 | 2.7 (1.7–4.3) | 32 | 20 | 2.3 (1.2–4.2) |
| ≥30 | 200 | 86 | 4.3 (3.0–6.1) | 131 | 57 | 4.6 (2.9–7.5) | 69 | 29 | 4.0 (2.3–7.0) |

[a]OR, odds ratio, adjusted for level of education (high school or less, college or above); CI, confidence interval.

included: demographic characteristics, adult height, weight at age 20, usual adult weight, maximum adult weight that was unrelated to pregnancy, lifetime use of tobacco and alcohol, usual dietary habits, lifetime occupational history, history of physician-diagnosed hypertension and other selected medical conditions, use of diuretics and other commonly prescribed drugs to control hypertension, use of prescription and non-prescription diet pills, and use of prescription and non-prescription analgesics. We listed 58 brand names of diuretics and antihypertensives, and 26 diet pills in the questionnaire (see Appendix), drugs representing all the common prescription medications in these respective categories marketed in the United States since the 1950s. A picture album of the named drugs was available to the respondent to assist in recall.

Regular use was defined as taking a listed brand name drug two or more times a week for 1 month or longer. We asked subjects the ages at first and last use, duration of use, usual dosage, and the primary reason for such use, including any other brand name diuretics, antihypertensives, or diet pills that were not listed in the questionnaire.

We attempted to verify self-reported usage of all prescription diuretics, antihypertensives and diet pills. All physicians named by study subjects were contacted to request information on dates of continuous care of the patients and details on the named prescriptions.

The formulations of each of the listed drugs as well as those volunteered by study subjects were established through numerous pharmaceutical sources, including the annually updated *Physician's Desk Reference*. Each class of drugs was then placed into major formulation categories; for example, diuretics were grouped as thiazides, furosemides or potassium-sparing diuretics. Antihypertensives were classified as beta blockers, central anti-adrenergic agents, neuronal depleting agents, angiotensin-converting enzyme inhibitors or vasodilators. Diet pills were categorized as amphetamines or other anorexic drugs. Age-specific exposure to a given drug was estimated from the subject's reported dose and duration of use at that age. Lifetime cumulative exposure to a specific class of compounds (in grams) was computed by summing age-specific exposures across all brand name drugs belonging to that class of compounds. Cumulative exposures were grouped into tertiles according to their distributions among control subjects.

Data were analysed using standard matched-pair methods (Breslow and Day, 1980). The associations of RCC with the various exposures were measured by odds ratios (ORs) and their corresponding 95% confidence intervals (CIs). Conditional logistic regression models were used to examine the univariate and multivariate relationships of RCC with various exposure variables, and to adjust for potential confounding factors including regular use of analgesics (M Gago-Dominguez et al, submitted for publication) and cigarette smoking (JM Yuan et al, submitted for publication), which were significant risk factors for RCC identified in the present study. The analysis of covariance methods (Winer, 1971) were used to compare levels of cumulative dosage of diuretics between cases and controls stratified by history of hypertension while controlling for age, level of education and obesity. ORs with two-sided $P$-values less than 0.05 are considered statistically significant. All $P$-values quoted are two-sided.

### RESULTS

There were 781 male and 423 female patients. The mean age at diagnosis of RCC was 58.8 years. Among patients, 1028 were non-Hispanic whites, 107 were Hispanic whites and 69 were blacks. Since 48% of cases vs 37% of control subjects did not attend college (OR = 0.6; 95% CI = 0.5–0.7), all subsequent analyses were adjusted for level of education.

The body mass index (BMI, defined as weight in kg divided by height in m squared, kg $m^{-2}$) was used as a marker of obesity. All three BMIs studied (i.e. BMI at age 20, maximum BMI and usual BMI) were significantly associated with risk of RCC and strengths of their associations were broadly similar. Results were also similar between men and women (Table 1).

A history of hypertension was significantly related to a 2.2-fold excess in RCC risk. Because advanced renal disease can lead to hypertension, we examined risk according to time interval between two diagnoses. There was little variation in risk between the subgroups. There was also no statistical difference in risk for RCC between treated and untreated hypertensive patients ($P = 0.11$) (Table 2).

In univariate analysis, significantly increased risks for RCC were noted among diabetics (OR = 1.6; 95% CI = 1.1–2.2) and stroke patients (OR = 2.1; 95% CI = 1.2–3.7). However, after adjustment for level of education, usual BMI and history of hypertension, the ORs reduced to 0.9 for diabetes and 1.2 for stroke, neither of which was statistically significant. No statistically significant association with RCC was observed for prior renal conditions such as renal stones, renal injury, renal infection or other renal disorders (data not shown).

Table 3 presents the combined effect of obesity and hypertension on risk of RCC. Regardless of hypertension status, the ORs increased with increasing usual BMI. Similarly, irrespective of level of usual BMI, hypertensive subjects had a roughly twofold increase in risk of RCC relative to normotensive subjects. Further adjustment for cigarette smoking, regular use of analgesics and regular use of amphetamines (see below) had little effect on the association.

© Cancer Research Campaign 1998

**Table 2** The effect of hypertension on risk of renal cell carcinoma

| Hypertension | Cases | Controls | OR[a] (95% CI) |
|---|---|---|---|
| No | 669 | 875 | 1.0 |
| Yes[b] | 535 | 329 | 2.2 (1.8–2.6) |
| Number of years since first diagnosis | | | |
| <5 | 55 | 40 | 2.1 (1.3–3.2) |
| 5–9 | 122 | 85 | 1.8 (1.4–2.5) |
| 10–19 | 188 | 96 | 2.6 (2.0–3.4) |
| 20–29 | 97 | 62 | 2.2 (1.6–3.2) |
| ≥30 | 63 | 40 | 2.0 (1.3–3.1) |
| Unknown | 10 | 6 | 1.8 (0.7–5.2) |
| Ever medically treated (by diuretics or antihypertensive drugs) | | | |
| No | 98 | 74 | 1.7 (1.2–2.3) |
| Yes | 437 | 255 | 2.3 (1.9–2.9) |

[a]OR, odds ratio, adjusted for level of education (high school or less, college or above); CI, confidence interval. [b]Diagnosed by physician.

**Table 3** The combined effect of obesity and hypertension on risk of renal cell carcinoma

| Usual body mass index (kg m$^{-2}$) | No hypertension | | | Hypertension | | |
|---|---|---|---|---|---|---|
| | Cases | Controls | OR[a] (95% CI) | Cases | Controls | OR[a] (95% CI) |
| <22 | 135 | 236 | 1.0 | 53 | 53 | 1.8 (1.1–2.8) |
| 22–<24 | 157 | 211 | 1.5 (1.1–2.1) | 90 | 58 | 3.4 (2.2–5.2) |
| 24–<26 | 165 | 212 | 1.5 (1.1–2.1) | 101 | 82 | 2.3 (1.6–3.4) |
| 26–<28 | 72 | 104 | 1.5 (0.98–2.2) | 92 | 66 | 2.8 (1.9–4.3) |
| 28–<30 | 60 | 63 | 1.8 (1.2–2.9) | 79 | 33 | 4.6 (2.9–7.5) |
| ≥30 | 80 | 49 | 3.2 (2.0–5.2) | 120 | 37 | 7.0 (4.4–11.3) |

[a]OR, odds ratio, adjusted for level of education (high school or less, college or above); CI, confidence interval.

Irregular use of diuretics was unrelated to risk for RCC (OR = 1.3; 95% CI = 0.7, 2.5 relative to never users), whereas regular users exhibited a 2.2-fold increased risk (95% CI = 1.8, 2.8). We examined the relationship between regular use of diuretics and RCC development by hypertensive status (Table 4). All hypertensive users indicated that the reason for taking diuretics was for hypertension control. Among the self-reported normotensive users, fluid/weight loss was the primary reason for using the 'water pill' (21 cases, 19 controls). The other main reason was for heart problems (four cases, three controls). Among normotensive subjects, there was no significant association between diuretic use and RCC risk. Furthermore, mean cumulative lifetime dose of diuretics in normotensive cases was similar to that in normotensive controls. Similarly, among hypertensive subjects, risk was unrelated to cumulative lifetime dose of diuretics. Results were similar when we repeated the analysis within subgroups of diuretics according to formulation [see Table 4 for thiazides and potassium-spacing diuretics; data for furosemides not shown, being based on smaller numbers (73 cases, 31 controls)].

Twenty-one cases and four controls had used spironolactone regularly, and all except one control reported a history of hypertension. Although the OR was relatively high for this agent (OR = 3.5; 95% CI = 1.1–10.8), there was no statistical difference in the risk of RCC between users of spironolactone and users of other diuretics, after adjustment for level of education and usual BMI ($P = 0.26$).

We also examined the association between non-diuretic medications used for hypertension control and RCC. Relative to non-users, no increased risk of RCC was observed among irregular users (OR = 1.0), whereas regular use was associated with a 1.8-fold increase in risk (95% CI = 1.4–2.2). Among self-reported normotensive subjects, 33 cases and 37 controls had used anti-hypertensives regularly (OR = 1.1; 95% CI = 0.6–1.8). The primary reasons for such use were heart problems (22 cases, 27 controls) and migraine headaches (six cases, four controls). Among hypertensive subjects, there was no significant difference in cumulative lifetime dose (in grams) of antihypertensives between cases and controls ($P = 0.13$). The results of analyses by subgroup of anti-hypertensives (beta blockers, central antiadrenergic agents, neuronal depleting agents, angiotensin-converting enzyme inhibitors, and vasodilators) were consistent with those based on the full data set.

A statistically significant 60% increased risk of RCC was noted in those who used diet pills regularly compared with those who did not. The effect was confined to subjects who used amphetamine-containing diet pills. We examined the association between RCC risk and amphetamines by dose level and by reason for use. There was a monotonic increase in risk by increasing maximum weekly dose of amphetamines ($P < 0.001$, linear trend test) after adjustment for level of education, usual BMI and history of hypertension. Reason for use of amphetamines had no influence on risk level (Table 5).

The multivariate relationship of RCC with usual BMI, hypertension and regular use of amphetamines was examined using a conditional logistic regression model that also included level of education, cigarette smoking and regular use of analgesics. Using BMI of < 22 as the reference category, adjusted ORs for RCC were 1.6 (95% CI = 1.2–2.0) for BMI of 22–<28, 2.1 (95% CI = 1.4–3.1)

© Cancer Research Campaign 1998

**Table 4** The relationship between use of diuretics and risk of renal cell carcinoma

| | Any diuretic | | | Thiazides | | | Potassium-sparing diuretics | | |
|---|---|---|---|---|---|---|---|---|---|
| | Cases | Controls | OR[a] (95% CI) | Cases | Controls | OR[a] (95% CI) | Cases | Controls | OR[a] (95% CI) |
| No use of diuretics | 846 | 1006 | 1.0 | 846 | 1006 | 1.0 | 846 | 1006 | 1.0 |
| Regular use[b] | 358 | 198 | 1.9 (1.6–2.4) | 320 | 181 | 1.9 (1.5–2.4) | 199 | 105 | 2.0 (1.6–2.7) |
| No hypertension | 25 | 24 | 1.2 (0.7–2.2) | 18 | 20 | 1.1 (0.6–2.1) | 11 | 11 | 1.2 (0.5–3.0) |
| Cumulative dose CM (g)[c,d] | | | | | | | | | |
| Low | 11 | 11 | 1.0 (0.4–2.5) | 6 | 9 | 0.8 (0.3–2.4) | 5 | 6 | 0.9 (0.2–3.2) |
| Medium | 5 | 5 | 1.6 (0.5–5.9) | 3 | 4 | 1.0 (0.2–4.5) | 3 | 3 | 1.7 (0.3–8.5) |
| High | 7 | 8 | 1.0 (0.3–2.8) | 6 | 7 | 1.0 (0.3–3.3) | 2 | 2 | 1.1 (0.1–8.5) |
| Adjusted mean CM (g) | 107 | 115 | | 82 | 91 | | 81 | 46 | |
| Two-sided *P*-value[e] | 0.85[e] | | | 0.85[e] | | | 0.29[e] | | |
| Hypertension | 333 | 174 | 2.0 (1.6–2.5) | 302 | 161 | 2.0 (1.6–2.5) | 188 | 94 | 2.1 (1.6–2.8) |
| Cumulative dose (g)[c,d] | | | | | | | | | |
| Low | 97 | 49 | 2.3 (1.6–3.4) | 52 | 35 | 1.9 (1.2–3.0) | 53 | 24 | 2.6 (1.5–4.3) |
| Medium | 100 | 56 | 1.8 (1.3–2.6) | 121 | 61 | 2.0 (1.4–2.8) | 58 | 31 | 2.1 (1.3–3.3) |
| High | 106 | 53 | 2.1 (1.4–3.0) | 99 | 48 | 2.2 (1.5–3.3) | 57 | 31 | 1.8 (1.1–3.0) |
| Adjusted mean CM (g) | 185 | 157 | | 125 | 92 | | 113 | 111 | |
| Two-sided *P*-value[e] | 0.32[e] | | | 0.19[e] | | | 0.95[e] | | |

[a] OR, odds ratio, adjusted for level of education (high school or less, college or above) and usual body mass index (kg m$^{-2}$); CI, confidence interval. [b]Defined as two or more times a week for 1 month or longer. [c]The sum may be slightly less than the total number of users because of the exclusion of subjects with missing values in cumulative dose. [d]Cumulative dose for low, medium and high levels, respectively, were: <43, 43–136, ≥137 for any diuretics; <17, 17–72, ≥73 for thiazides and <28, 28–100, ≥100 for potassium-sparing diuretics. [e]Analysis of covariance with cumulative dosage of diuretics and level of education (high school or less, college or above) as main effects, and age and usual body mass index (kg m$^{-2}$) as regression covariates.

**Table 5** The relationship between use of diet pills and risk of renal cell carcinoma

| | Cases | Controls | OR[a] (95% CI) |
|---|---|---|---|
| Regular use of any diet pill[b] | | | |
| No | 1028 | 1094 | 1.0 |
| Yes | 176 | 110 | 1.6 (1.2–2.1) |
| Amphetamines only | 89 | 47 | 2.0 (1.3–2.9) |
| Non-amphetamines only | 57 | 49 | 1.1 (0.7–1.7) |
| Combined use | 30 | 14 | 2.0 (0.96–4.0) |
| Regular use of amphetamine-containing diet pills[b] | | | |
| No | 1085 | 1143 | 1.0 |
| Yes | 119 | 61 | 2.0 (1.4–2.8) |
| Maximum weekly dose of amphetamine (mg)[c] | | | |
| 1–37.5 | 25 | 18 | 1.5 (0.7–2.9) |
| 37.6–75.0 | 33 | 22 | 1.9 (1.04–3.4) |
| ≥75.1 | 55 | 18 | 2.6 (1.5–4.6) |
| Reason for use[c] | | | |
| Weight reduction | 68 | 31 | 2.1 (1.3–3.3) |
| Other | 45 | 28 | 1.8 (1.1–3.0) |

[a] OR, odds ratio, adjusted for level of education (high school or less, college or above), usual body mass index (kg m$^{-2}$), and history of hypertension; CI, confidence interval. [b]Defined as two or more times a week for 1 month or longer. [c]The sum may be slightly less than the total number of users due to the exclusion of subjects with missing values in the analysis.

for BMI of 28–<30, and 3.4 (95% CI = 2.3–4.9) for BMI of ≥30. The adjusted OR was 1.9 (95% CI = 1.6–2.3) for a history of hypertension and 1.7 (95% CI = 1.2–2.5) for regular use of amphetamines.

Analyses that excluded the 98 pairs who were not matched on race did not materially change the associations of RCC risk with obesity, history of hypertension, and use of diuretics, antihypertensives or diet pills.

Of the 778 prescription diuretics reported by study subjects (520 by cases, 258 by controls), physician response rate was similar between cases (35%) and controls (40%). Brand name concordance rate was 62% in cases and 62% in controls. Of the 129 physician responses with dosage information, concordance rate was 94% in cases and 91% in controls.

Study subjects reported 873 individual non-diuretic antihypertensive prescriptions (552 by cases, 321 by controls). Physician response rate was similar between cases (39%) and controls (38%). Brand name concordance rate was equally high among cases (67%) and controls (63%). Of the 182 physician responses with dosage information, concordance rate was 88% in cases and 86% in controls.

Study subjects reported 295 individual prescription diet pills (182 by cases, 113 by controls), but only a few of these could be validated. Sixty-three per cent of these diet pills were used at least 20 years ago, so either subjects could not recollect the name of physician who prescribed the diet pills, the named physician could not be located or the patient's medical records could not be found. Moreover, many patients did not get their diet pills through the conventional healthcare system.

### DISCUSSION

To our knowledge, the present study is the largest case–control study of RCC ever conducted on a single, geographically defined study population. Our study was specifically designed to examine the association of RCC with use of diuretics, antihypertensives and diet pills, as well as with the conditions (hypertension and obesity) that call for the use of these drugs. Diagnoses of all cases were histologically confirmed; 14% clear cell carcinoma; 2% granular cell carcinoma; and the remaining 84% renal cell carcinoma without cell type specification. The names of all commonly used diuretics, antihypertensives, and diet pills were explicitly listed in the study questionnaire. In a separate section of the questionnaire, a detailed medical history that included physician-diagnosed hypertension was collected. Comparison of responses from these two sections of the questionnaire revealed a remarkable degree of consistency in recalled information from subjects. Moreover, RCC patients did not differ from control subjects in the degree of consistency between self-reported and validated information on use of diuretics and antihypertensives, even although only a relatively small percentage of medications were validated. Most importantly, the large sample size of this study allows for the effects of diuretics and antihypertensives to be investigated among those who were prescribed these drugs for reasons other than hypertension control, and to explore differences in risk between treated and untreated hypertensives.

The present data do not implicate use of diuretics as an independent risk factor for RCC. Since our first observation of an association between diuretic use and RCC (Yu et al, 1986), a number of case–control studies have substantiated this relationship (McLaughlin et al, 1988; Finkle et al, 1993; Kreiger et al, 1993; Hiatt et al, 1994; Weinmann et al, 1994). These later studies, however, all suffer from one or more of the major flaws present in our earlier investigation (crude exposure assessment, inclusion of proxy interviews, small sample size and limited analysis to separate treatment effects from their indications). Heath et al (1997) recently reported, from a cohort study of 1.2 million adult Americans, that use of diuretics was associated with a 60% increased risk of death from RCC in women but not in men. However, this result was not adjusted for a history of hypertension and obesity. Recently, McLaughlin et al (1995) described a large, multicentre study involving 1732 renal cell cancer patients and 2309 controls, which circumvented many of the design flaws of the earlier studies. The investigators noted no association between diuretic use and risk of RCC after adjustment for study centre, age, sex, BMI, cigarette smoking and history of hypertension.

Spironolactone, a potassium-sparing diuretic, has been found to be tumorigenic in experimental animals (BeDell, 1996). Ron et al (1987) reported a non-significant association with thyroid cancer based on two positive cases and one positive control. We found no clear evidence that the compound is a renal carcinogen.

There is no a priori reason to suspect that non-diuretic antihypertensives (beta blockers, central antiadrenergic agents, neuronal depleting agents, angiotensin-converting enzyme inhibitors and vasodilators) are renal carcinogens. However, McLaughlin et al (1995) recently reported that long-term use (5 or more years) of these drugs was associated with a significant increase in risk of RCC independent of a history of hypertension, although no dose–response relationship was observed with cumulative dosage of any of the major classes of antihypertensives. Heath et al (1997) showed that use of antihypertensives was associated with a statistically significant increase in risk of death from RCC among female participants of the American Cancer Society Cohort Study. However, the effects of antihypertensives and history of hypertension could not be disentangled and no statistically significant dose–response relation between lifetime duration of use or dosage per month and risk of RCC was observed. We detected no positive association between regular use of such drugs and RCC risk after adjustment for hypertension status. The fact that similar risks of RCC were observed between never- and ever-treated hypertensives also argues against the non-diuretic antihypertensives having substantial independent effects on RCC risk.

Several studies have reported a high risk of RCC in obese individuals, especially women (Yu et al, 1986; Mellemgaard et al, 1995; Chow et al, 1996) and the present study confirms this in both men and women.

Hypertension is clearly a major independent risk factor for RCC in the present study. The association between hypertension and RCC is unlikely to be the consequence of renal cancer as subjects who were diagnosed with hypertension 20 or more years before cancer diagnosis still experienced a statistically significant two-fold elevation in risk. This is not a novel finding as numerous case–control and cohort studies have also observed this relationship (Raynor et al, 1981; Grove et al, 1991; Kreiger et al, 1993; Heath et al, 1997). Diabetes and stroke, which are known to be related to obesity and hypertension, were also associated with RCC risk in our study, but these associations disappeared after adjustment for obesity and history of hypertension.

Experimental work in rodents has demonstrated that obesity and hypertension can lead to renal glomerulosclerosis and tubulointerstitial cell proliferation (Keane et al, 1993; Mai et al, 1993; O'Donnell et al, 1993; Eng et al, 1994). As far as we know, none of these rodent models included tumour development as an outcome measurement, and their relevance to human renal carcinogenesis is uncertain.

Amphetamine use was associated with increased risk of RCC in the present study. A dose–response relation between maximum weekly dose and risk of RCC was observed in both men and women. In case recall bias might explain this association, we indirectly addressed this issue in an on-going study of bladder cancer in Los Angeles, which uses the same questionnaire regarding medication use as the RCC study. A total of 1232 bladder cancer patients and an equal number of age-, sex- and race-matched neighbourhood controls were included in this analysis. The bladder cancer patients were slightly younger (aged 25–69 years) than the RCC patients. The exposure profiles of amphetamines among the controls in both the bladder cancer study and the present study were similar. There were 81 (6.6%) bladder cancer patients and 78 (6.3%) control subjects who had ever used amphetamine-containing diet pills regularly. The relative risk of bladder cancer for regular use of amphetamines was 0.9 (95% CI = 0.6–1.2) and

© Cancer Research Campaign 1998

there was no dose–response relation between maximum weekly dose of amphetamines and risk of bladder cancer ($P = 0.67$, linear trend test). In a recent multicentre study, Mellemgaard et al. (1995) also reported a statistically significant increase in risk of RCC among amphetamine users, but with no evidence of increasing risk with increasing cumulative dose or duration of use. For a drug such as a diet pill with a usage pattern that is typically sporadic and intermittent, self-reported duration of use is likely to be inaccurate. The role of amphetamine use in RCC aetiology requires further investigation.

In summary, the present study has demonstrated that chronic obesity and a history of hypertension are important risk factors for RCC in Los Angeles, California. There was no strong evidence that use of diuretics and antihypertensives are independently related to RCC development. Regular use of amphetamines may be associated with an increased risk of RCC; however, the fraction of cases possibly related to this exposure is small (population-attributable risk = 5%).

### ACKNOWLEDGEMENT

This study was supported by grants P01 CA17054 and R35 CA53890 from the United States National Cancer Institute, Bethesda, MD. We thank Ms Susan Roberts, Mr Roger Mathison and Ms Kazuko Arakawa of the University of Southern California for their assistance in data collection and management. We also thank Dr Kenneth K Chan of the Ohio State University for his assistance in classifying diuretics and antihypertensive medications.

### APPENDIX

Diuretics, aldactazide, aldactone, diuril, dyazide, enduron, esidrix, furosemide, hydrochlorothiazide, hydrodiuril, hygroton, lasix, metahydrin, oretic, and zaroxolyn; antihypertensives, aldomet, apresoline, capoten, catapres, corgard, hydralazine, inderal, lopressor, minipress, procardia, rau-sed, reserpine, serpasil and tenormin; and diuretic/antihypertensive combination drugs, adoril, esimil, hydropres and serapes.

Amphetamine-containing diet pills, benzedrine, biphetamine, dexamyl, dexedrine, eskatrol and obetrol; and non-amphetamine containing diet pills, adipex-P, anorexin, appedrine, bacarate, bontril PDM, control, dexatrim, didrex, fastin, ionamin, plegine, pondimin, pre-sate, preludin, prolamine, sanorex, statobex, tenuate, tepanil and voramil.

### REFERENCES

BeDell LS (1996) *Physicians GenRx: the Complete Drug Reference*, pp.II. 1902–II.1904. Mosby-Year Book: St Louis, MO, USA.

Bernstein L and Ross RK (1991). *Cancer in Los Angeles County. A Portrait of Incidence and Mortality 1972–1987*. University of Southern California: Los Angeles, CA.

Breslow NE and Day NE (1980) *Statistical Methods in Cancer Research, Volume I: The Analysis of Case–Control Studies*. IARC Scientific publications 32, IARC: Lyon, France.

Chow WH, McLaughlin JK, Mandel JS, Wacholder S, Niwa S and Fraumeni Jr JF (1996) Obesity and risk of renal cell cancer. *Cancer Epidemiol Biomarkers Prev* **5**: 17–21

Eng E, Veniant M, Floege J, Fingerle J, Alpers CE, Menard J, Clozel J-P and Johnson RJ (1994) Renal proliferative and phenotypic changes in rats with two-kidney, one-clip Goldblatt hypertension. *Am J Hypertens* **7**: 177–185

Finkle WD, McLaughlin JK, Rasgon SA, Yeoh HH and Low JE (1993) Increased risk of renal cell cancer among women using diuretics in the United States. *Cancer Caus Contr* **4**: 555–558

Gago-Dominguez M, Yuan J-M, Castelao JE, Ross RK and Yu MC. Regular use of analgesics is a risk factor for renal cell carcinoma. *J Natl Cancer Inst* (submitted)

Grove JS, Nomura A, Severson RK and Stemmermann GN (1991) The association of blood pressure with cancer incidence in a prospective study. *Am J Epidemiol* **134**: 942–947

Heath CW Jr, Lally CA, Calle EE, McLaughlin JK and Thun MJ (1997) Hypertension, diuretics, and antihypertensive medications as possible risk factors for renal cell cancer. *Am J Epidemiol* **145**: 607–613

Hiatt RA, Tolan K and Quesenberry CP Jr (1994) Renal cell carcinoma and thiazide use: a historical, case-control study (California, USA). *Cancer Caus Contr* **5**: 319–325

Keane WF, Kasiske BL, O'Donnell MP and Kim Y (1993) Hypertension, hyperlipidemia, and renal damage. *Am J Kidney Dis* **21**: 43–50

Kreiger N, Marret LD, Dodds L, Hilditch S and Darlington GA (1993) Risk factors for renal cell carcinoma: results of a population-based case-control study. *Cancer Caus Contr* **4**: 101–110

McLaughlin JK, Blot WJ, Fraumeni JF Jr (1988) Diuretics and renal cell cancer. *J Natl Cancer Inst* **80**: 378

McLaughlin JK, Chow WH, Mandel JS, Mellemgaard A, McCredie M, Linblad P, Schlehofer B, Pommer W, Niwa S and Adami H-O (1995) International renal-cell cancer study. VIII. Role of diuretics, other antihypertensive medications and hypertension. *Int J Cancer* **63**: 216–221

Mai M, Geiger H, Hilgers KF, Veelken R, Mann JFE, Dammrish J and Luft FC (1993) Early interstitial changes in hypertension-induced renal injury. *Hypertension* **22**: 754–765

Mellemgaard A, Lindblad P, Schlehofer B, Bergstrom R, Mandel JS, McCredie M, McLaughlin JK, Niwa S, Odaka N, Pommer W and Olsen JH (1995) International renal-cell cancer study. III. Role of weight, height, physical activity, and use of amphetamines. *Int J Cancer* **60**: 350–354

O'Donnell MP, Kasiske BL, Kim Y, Schmitz PG and Keane WF (1993) Lovastatin retards the progression of established glomerular disease in obese Zucker rats. *Am J Kidney Dis* **22**: 83–89

Raynor WJ Jr, Shekelle RB, Rossof AH, Maliza C and Paul O (1981) High blood pressure and 17-year cancer mortality in the Western Electric Health Study. *Am J Epidemiol* **113**: 371–377

Ron E, Kleinerman RA, Boice JD Jr, Livolsi VA, Flannery JT and Fraumeni JF Jr (1987) A population-based case-control study of thyroid cancer. *J Natl Cancer Inst* **79**: 1–12

Weinmann S, Glass AG, Weiss NS, Psaty BM, Siscovick DS and White E (1994) Use of diuretics and other antihypertensive medications in relation to the risk of renal cell cancer. *Am J Epidemiol* **140**: 792–804

Winer BJ (1971) *Statistical Principles in Experimental Design*, (2nd edn), pp. 149–260. McGraw Hill: New York

Yu MC, Mack TM, Hanisch R, Cicioni C and Henderson BE (1986) Cigarette smoking, obesity, diuretic use, and coffee consumption as risk factors for renal cell carcinoma. *J Natl Cancer Inst* **77**: 351–356

Yuan J-M, Castelao JE, Gago-Dominguez M, Yu MC and Ross RK Tobacco use in relation to renal cell carcinoma. *Cancer Epidemiol Biomarkers Prev* (submitted)

© Cancer Research Campaign 1998